# Fulvestrant up regulates *UGT1A4* and *MRP*s through ERα and c-Myb pathways: a possible primary drug disposition mechanism

**DOI:** 10.1186/2193-1801-2-620

**Published:** 2013-11-20

**Authors:** Vineetha K Edavana, Rosalind B Penney, Aiwei Yao-Borengasser, Suzanne Williams, Lora Rogers, Ishwori B Dhakal, Susan Kadlubar

**Affiliations:** Division of Medical Genetics, College of Medicine, University of Arkansas for Medical Sciences, 4301 W. Markham, #580, Little Rock, AR 72205 USA; Department of Environmental and Occupational Health, College of Public Health, Little Rock, AR 72205 USA

**Keywords:** Fulvestrant, Anastrozole, *UGT1A4*, *MRP*s, *ERα*, *c-Myb*

## Abstract

Fulvestrant (Faslodex™) is a pure antiestrogen that is effective in treating estrogen receptor-(ER) positive breast cancer tumors that are resistant to selective estrogen receptor modulators such as tamoxifen. Clinical trials investigating the utility of adding fulvestrant to other therapeutics have not been shown to affect cytochrome P450-mediated metabolism. Effects on phase II metabolism and drug resistance have not been explored. This study demonstrates that fulvestrant up regulates the expression of UDP glucuronosyltransferase 1A4 (*UGT1A4*) >2.5- and >3.5-fold in MCF7 and HepG2 cells, respectively. Up regulation occurred in a time- and concentration-dependent manner, and was inhibited by siRNA silencing of ERα. Fulvestrant also up regulates multidrug resistance-associated proteins (MRPs). There was an up regulation of *MRP*2 (1.5- and 3.5-fold), and *MRP*3 (5.5- and 4.5-fold) in MCF7 and HepG2 cell lines, respectively, and an up regulation of *MRP1* (4-fold) in MCF7 cells. *UGT1A4* mRNA up regulation was significantly correlated with UGT1A4 protein expression, anastrozole glucuronidation, *ERα* mRNA expression and *MRP* mRNA expression, but not with ERα protein expression. Genetic variants in the *UGT1A4* promoter (-163A, -217G and -219T) reduced the basal activity of *UGT1A4* by 40-60%. *In silico* analysis indicated that transcription factor *c-Myb* binding capacity may be affected by these variations. Luciferase activity assays demonstrate that silencing *c-Myb* abolished UGT1A4 up regulation by fulvestrant in promoters with the common genotype (-163G, -217 T and -219C) in MCF7 cells. These data indicate that fulvestrant can influence the disposition of other UGT1A4 substrates. These findings suggest a clinically significant role for *UGT1A4* and *MRP*s in drug efficacy.

## Introduction

Fulvestrant (Faslodex™; ICI 182,780) belongs to a novel class of endocrine agents for the treatment of breast cancer (Howell et al. 2000;Osborne et al. 2004). Fulvestrant is a pure antiestrogen which is effective in treating estrogen receptor (ER) positive tumors that are resistant to selective estrogen receptor modulators (SERMs) such as tamoxifen This compound differs significantly from tamoxifen in its mode of action, which is through promoting the rapid degradation of the ER. Fulvestrant shows no estrogen agonist activity, and thus has been regarded as an important improvement in breast cancer therapy (Morris and Wakeling 2002;Howell et al. 2002;Osborne et al. 2002). It is thought that co-administration of fulvestrant with other therapeutics may be beneficial, and clinical trials investigating this are being conducted (Group 2004;AstraZeneca 2012). However, the effect of co-administration on phase II drug metabolism and drug disposition has not yet been reported.

The phase II biotransformation system comprises an array of enzymes that incorporate a hydrophilic group into hydrophobic molecules, thereby increasing solubility and potentially decreasing the toxicity of the original target. Altered rates of metabolism can affect systemic availability and elimination half-life of xenobiotics. This can affect toxicity or therapeutic effect, and may result in undesirable drug-drug interactions. A better understanding of simultaneous regulation of metabolism and disposition may help prevent these undesirable effects.

Fulvestrant is primarily metabolized by cytochrome P450 enzymes (CYPs) and phase II enzymes like sulfotransferases (SULTs) and UDP-glucuronosyltransferases (UGTs). Fulvestrant has been shown to be glucuronidated by human recombinant UGT1A1, UGT1A3, UGT1A4 and UGT1A8 enzymes. Kinetic analysis has revealed that UGT1A4 displays the highest affinity for fulvestrant, and that UGT1A3 and UGT1A4 display the highest catalytic efficiency for fulvestrant glucuronidation (Chouinard et al. 2006;Starlard-Davenport et al. 2010).

Previous studies from this laboratory report that genetic variations in *UGT1A4* have a potential role in inter-individual variability in anastrozole glucuronidation (Edavana et al. 2013). These findings indicate that alterations in UGT1A4 may significantly affect the glucuronidation rates of fulvestrant. Thus variations may also affect metabolism and disposition of drugs that are co-administered with fulvestrant and which are substrates for UGT1A4.

Drug disposition can be affected not only by factors such as metabolic genes, but also transporter proteins (Bock et al. 2000;Catania et al. 2004). Multidrug resistance-associated proteins (MRPs) are a family of ATP-dependent transporters that exhibit elevated expression levels in tumor cells. MRPs are preferentially localized in the apical membrane of hepatocytes, renal tubular cells and enterocytes (Catania et al. 2004). They are involved in the secretion of a large number of conjugated compounds, and most MRP substrates are conjugated derivatives of endogenous compounds, drugs and carcinogens (Keppler et al. 1997). Thus, MRPs may act coordinately with phase II enzymes to eliminate these compounds from the body. In the present study, the effects of fulvestrant on phase II metabolism regulation, drug disposition and interactions with other therapeutics are explored in breast cancer and liver cancer cell lines.

## Materials and methods

### Chemicals and reagents

Fulvestrant was provided by AstraZeneca Pharmaceuticals (Macclesfield, Cheshire, UK). Anastrozole (2,2′-[5-(1H-1,2,4-triazol-1-ylmethyl)-1,3-phenylene]bis (2-methylpropanenitrile) was obtained from Toronto Research Chemicals, Inc. (Toronto, Canada). Alamethicin and UDP glucuronic acid (UDPGA) were purchased from Sigma-Aldrich (St. Louis, MO). Baculovirus-expressed human UGT1A4 was purchased from BD Gentest Corp. (Woburn, MA). *ERα* siRNA(h), *c-Myb* siRNA (h), ERα, c-Myb and UGT1A4 primary and secondary antibodies were purchased from Santa Cruz Biotechnology, Inc. (Santa Cruz, CA). Actin primary antibody was purchased from Sigma-Aldrich (St. Louis, MO) Chemiluminescence reagents were obtained from GE Healthcare (Piscataway, NJ). All other reagents were of HPLC grade or of the highest grade commercially available.

### Cell culture

MCF7 cells from American Type Culture Collection were maintained in RPMI 1640 supplemented with 10% fetal bovine serum, 2 mM L-glutamine, 6 ng of bovine insulin/ml, 100 units of penicillin/ml, 100 μg of streptomycin/ml, and 1% minimal essential medium nonessential amino acids. HepG2 cells (a kind gift from Yevgeniy Apostolov, UAMS, AR) were cultured in complete Dulbecco’s modified Eagle’s medium (MediaTech, Inc., Manassas, VA) supplemented with 10% fetal bovine serum (Gemini, Woodland, CA), 2 mM L-glutamine, 100 units of penicillin/ml, 100 μg of streptomycin/ml, and 1% minimal essential medium nonessential amino acids. The media were changed 3 days before each experiment to estrogen-free media, i.e. complete Dulbecco’s modified Eagle’s medium containing charcoal/dextran-stripped fetal bovine serum (Gemini) and no phenol red.

### Transfection of *ERα* and *c-Myb* siRNA

*ERα* and *c-Myb* siRNAs were transfected into cells according to manufacturer’s protocol. The final concentration of inhibitor was 100 nM. After 48 h, cells were harvested, total RNA was isolated, and *ERα* and *c-Myb* expressions were measured by RT-PCR according to manufacturer’s protocol (see below).

### Quantitative real-time PCR

Total RNA was isolated using TRIzol (Invitrogen, Carlsbad, CA), and was used as a template for cDNA synthesis with Superscript II (Invitrogen, Grand Island, NY). Quantitative RT-PCR was performed using a Prism 7900HT Sequence Detection System and SYBR Green PCR Master Mix (Applied Biosystems, Foster City, CA). Gene specific primers, annealing temperature and cycle numbers for *UGT1A4*, *ERα*, *MRP*1, *MRP*2 and *MRP*3 have been described previously (Edavana et al. 2013;Ros et al. 2003;Walton et al. 2009). The dissociation curves for each reaction were examined to ensure amplification of a single PCR product in the reaction. The -fold change in mRNA levels was determined after normalizing the gene expression levels to those of β-actin (2^-ΔΔCt^ method) as described previously (Schmittgen and Livak 2008). Taqman gene expression assay (ABI, Foster city, CA) was performed to assess *c-Myb* expression.

### Transient transfection of *UGT1A4* promoter constructs and luciferase activity assay

A *UGT1A4* promoter construct of 1.5 kb was inserted upstream of the luciferase reporter gene in the pLightSwitch_Prom vector (SwitchGear Genomics, Menlo Park, CA). Variant alleles were generated with the QuikChange® Site-Directed Mutagenesis Kit (Stratagene, La Jolla, CA). Transient transfections of reference and variant promoter constructs (100 ng) were performed according to manufacturer’s protocol using Fugene HD (Promega, Sunnyvale, CA). After 24 hrs cells were treated either with fulvestrant (10 nM) or ethanol (vehicle, 0.1%) for 48 hours. Luciferase activity was assessed with the LightSwitch Luciferase assay system (SwitchGear Genomics) following the manufacturer’s protocol. Results were analyzed by normalizing luciferase in transfected cells to cells transfected with the empty pLightSwitch_Prom vector. To determine the effect of transcription factor *c-Myb* on *UGT1A4* promoter expression, MCF7 cells transfected with the variant promoter construct were treated with *c-Myb* siRNA before fulvestrant treatment, and luciferase activity was assessed.

### Preparation of cytosol microsomes from cell lines

Solubilized microsomal protein was prepared as described previously (Sirois et al. 1992;Muller-Decker et al. 1995) with minor modification. Briefly, subconfluent growth-arrested cells were homogenized on ice in Tris-EDTA-diethyldithiocarbamic (TED) buffer [50 mM Tris HCI (pH 8.0), 10 mM EDTA, and 1 mM diethyldithiocarbamic acid] containing 2 mM octyl glucoside. Cells were then centrifuged at 100,000 × g for 1 h at 4°C. The crude pellets were sonicated in TED sonication buffer [20 msi; Tris-HCI (pH 8.0), 50 mM EDTA, and 0.1 mM diethyldithiocarbamic acid] containing 45 mM octyl glucoside. The sonicates were centrifuged at 13,000 × g at 4°C, and the recovered supernatants containing the cytosolic microsomes were stored at -80°C until the assays were performed.

### Western blot

Cell lysates were separated by 12% SDS-polyacrylamide gel electrophoresis. Proteins were transferred to a polyvinylidene fluoride membrane and probed with anti-ERα, anti-c-Myb and anti-UGT1A4 antibodies according to manufacturer’s protocol. Membranes were then incubated with secondary antibody for 1 h before chemiluminescence detection using SuperSignal West Femto Maximum Sensitivity Substrate (Pierce, Rockford, IL). Actin was detected as a loading control. Images were collected and analyzed using the FluorChem™ SP imager with Alpha Ease FC (FluorChem™ SP) software (San Leandro, CA).

### *In silico* SNP analysis

The *in silico* program, is-rSNP, was utilized to explore binding capacity changes in the promoter containing variant sequences. The program utilizes JASPAR and TRANSFAC to access the position weight matrix, and uses this and a “sliding window approach” to create scores and p-values that relate to transcription factor binding capacity (Macintyre et al. 2010).

### Glucuronidation of anastrozole using microsomes isolated from MCF7 and HepG2 cell lines

The glucuronidation of anastrozole was measured in microsomes prepared from MCF7 and HepG2 cell lines. Enzymatic assays were performed according to standard procedure described previously (Benoit-Biancamano et al. 2009;Kamdem et al. 2010).

### Statistical analysis

Student’s t-tests were used to compare baseline and treatment measurements within a group. Pearson’s correlation coefficients were used to describe the linear association between variables. All data from samples were expressed as mean ± SEM. Statistical significance was set at p < 0.05.

## Results

### Induction of *UGT1A4* expression and activity by fulvestrant

**Figure 1:**
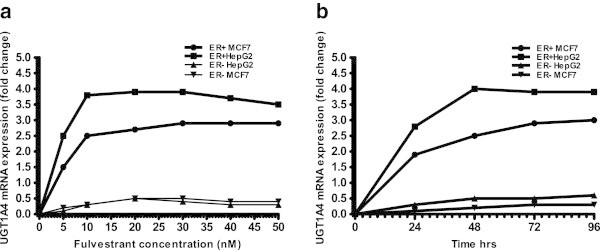
**Up regulation of** ***UGT1A4*** **mRNA expression in MCF7 and HepG2 cell lines treated with fulvestrant. (a)** MCF7 and HepG2 cells ± transfection with ERα siRNA were treated with various concentrations of fulvestrant before UGT1A4 gene expression was measured. All data was normalized to β-actin. **(b)** MCF7 and HepG2 cells were transfected as in (A), and then all were treated with 10 nM fulvestrant and analyzed for UGT1A4 expression at various time points.

To examine the effect of fulvestrant on the expression of *UGT1A4*, ERα + MCF7 and HepG2 cells were used. When concentration studies were performed, both cell lines showed strong sensitivity towards 10 nM fulvestrant. Cells were pre-treated with 1 nM fulvestrant for 30 days before experiments to decrease sensitivity and increase longevity during subsequent fulvestrant treatment. Cells were treated with different concentrations (0, 5, 10, 20, 30, and 50 nM) of fulvestrant for 48 hours. *UGT1A4* was up regulated by fulvestrant treatment, and the expression of UGT1A4 essentially plateaus at concentrations higher than 10 nM (Figure 1a). After treatment with 10 nM fulvestrant, cells were harvested at different time points (0, 24, 48, 72 and 96 hrs) and mRNA levels were determined by qRT-PCR. Treatment with fulvestrant up regulated *UGT1A4* in a time-dependent manner. Endogenous mRNA expression levels of *UGT1A4* peaked at 72 hours (>2.5-fold) in MCF7 cells, and at 48 hours (>3.5-fold) in HepG2 cells (Figure 1b). To determine if the regulation of UGT1A4 is mediated by the ER, siRNA silencing of *ERα* was performed before repeating these experiments, which abolished *UGT1A4* up regulation upon fulvestrant treatment (Figure 1a and b).

UGT1A4 protein levels were measured by Western blot and anastrozole glucuronidation activities were measured by mass spectrometry in MCF7 and HepG2 cells with and without *ERα* siRNA transfection before treatment. Treatments were with 10 nM fulvestrant, and data was collected over several time points. *UGT1A4* mRNA was correlated with UGT1A4 protein expression (r = 0.969 (MCF7) and 0.999 (HepG2), p < 0.01 (both)) and anastrozole glucuronidation activity (r = 0.875, p < 0.05 (MCF7), r = 0.961, p < 0.01 (HepG2); Table 1).

**Table 1:** **Correlation of UGT1A4, MRP and ERα in HepG2 and MCf7 Cell lines: Correlation analysis of MCF7/HepG2** ***UGT1A4*** **mRNA expression level with its own UGT1A4 protein, Anastrozole glucuronidation,** ***ERα*** **mRNA, ERα protein,** ***MRP*** **1,** ***MRP*** **2 and** ***MRP*** **3**

	MCF-7 ***UGT1A4*** mRNA	HepG2-***UGT1A4*** mRNA
MCF7/HepG2 UGT1A4 protein	0.969**	0.999**
MCF7/HepG2 Anastrozole glucuronidation	0.875*	0.961**
MCF7/HepG2 *ERα*-mRNA	0.915*	0.923**
MCF7/HepG2 ERα-protein	0.760*	0.753
MCF7/HepG2 *MRP*1	0.972**	NA
MCF7/HepG2 *MRP*2	0.906*	0.948**
MCF7/HepG2 *MRP*3	0.983**	0.967**

**p < 0.01; *p < 0.05.

### Correlation of *UGT1A4* mRNA with *ERα* mRNA and ERα protein levels

**Figure 2:**
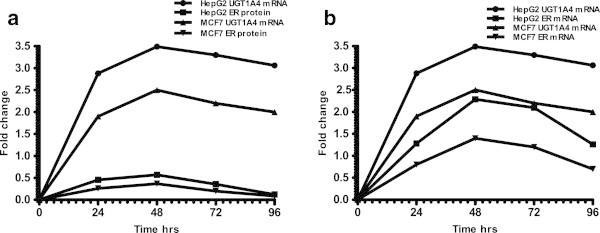
**Correlation of UGT1A4 mRNA with ER expression.** Correlation of UGT1A4 mRNA with ER protein **(a)** and with ER mRNA **(b)** in HepG2 and MCF7 cell lines treated with fulvestrant. Cells were treated with 10 nM fulvestrant. UGT1A4 and ER mRNA and protein expressions were measured at various time points.

To confirm that *UGT1A4* expression was partially mediated through the ER pathway, *ERα* mRNA and protein expression levels were measured and correlated to *UGT1A4* mRNA expression levels. (Figure 2a and b)*UGT1A4* mRNA expression levels correlated with *ERα* mRNA (r = 0.915, p < 0.05 for MCF; r = 0.923, p < 0.01 for HepG2), but not with ERα protein levels (r = 0.760, p > 0.05 for MCF7; r = 0.753, p > 0.05 for HepG2) in both cell lines (Table 1). These results indicate that fulvestrant induces *UGT1A4* expression at least partially through an ERα-mediated mechanism.

### Transcriptional activation of *UGT1A4* by fulvestrant

**Figure 3:**
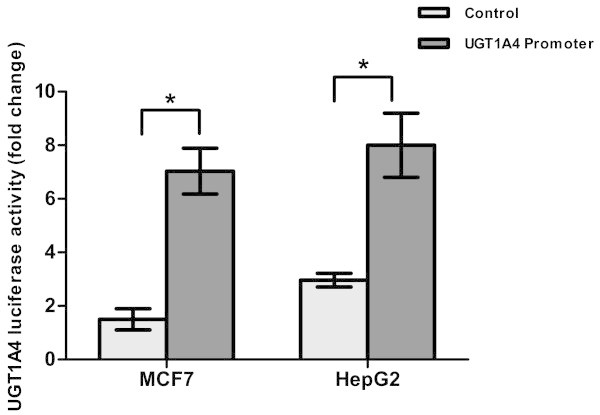
***UGT1A4*** **luciferase activity measured in MCF7 and HepG2 cell lines.** Cells were transfected either with empty vector or *UGT1A4* reference promoter. Then treated with 10 mM fulvestrant and luciferase activity was measured *p value < 0.01.

To study the mechanism of regulation of *UGT1A4* by fulvestrant, transcriptional activity modulation by the *UGT1A4* promoter was assessed by transient transfections in MCF7 and HepG2 cell lines. Each cell line was transfected with either pLightSwitch-*UGT1A4* or the empty pLightSwitch_Prom vector. The transfected cells were then treated either with fulvestrant or with the ethanol vehicle, and luciferase activities were determined. Upon treatment with fulvestrant, MCF7 and HepG2 cells transfected with pLightSwitch_*UGT1A4* exhibited >5-fold (p < 0.01) increase in luciferase activity compared with empty promoter-treated cells (Figure 3).

**Figure 4:**
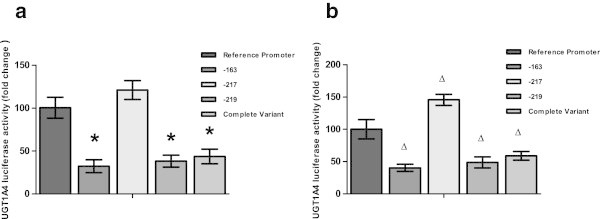
**Promoter variant luciferase activity in various cell lines**. Promoter variant luciferase activity measured in MCF7 **(a)** and HepG2 **(b)** cells. Cells were transfected either with UGT1A4 common allele promoter or with promoters with single variants at -163, -217, and -219 and with a promoter with all three variant (complete variant). Cells were then treated with 10 mM fulvestrant and luciferase activity was measured, and is displayed as percentage difference * p value < 0.01, Δ p value ≤ 0.01.

According to previous reports by this lab, *UGT1A4* promoter SNPs -163G > A, -217 T > G and -219C > T have been associated with inter-individual variability in enzymatic activity in human liver microsomes (Edavana et al. 2013). In this study, similar results were seen with fulvestrant treatment. The -163G > A variant or the -219C > T variant in either or both alleles reduced the basal luciferase activity by 40-50% (p < 0.01) and 30-40% (p ≤ 0.01) respectively in MCF7 and HepG2cells. The -217 T > G variant in either or both alleles increased the basal luciferase expression by 20-50% (p ≤ 0.01) in both cell lines. The complete variant haplotype (-163A, -217G and -219 T) reduced control luciferase activity by 40-60% (p ≤ 0.01) from the reference promoter in both cell lines (Figure 4).

**Figure 5:**
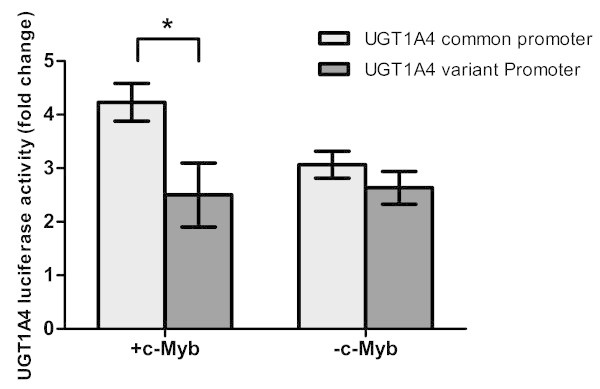
**Luciferase activity in MCF7 cells +/-** ***c-Myb*** **transfected with** ***UGT1A4*** **common or variant promoters.** Cells were transfected either with *UGT1A4* promoter constructs containing all common alleles or *UGT1A4* promoter constructs containing all variant alleles in locations -163, -217 and -219. Cells were treated with 10 mM fulvestrant and luciferase activity was measured, and is displayed as fold-change *p value = 0.01.

*In silico* analysis using is-rSNP revealed that the *UGT1A4* -163 variant is associated with changes in the *c-Myb* transcription factor binding site. MCF7 expresses *c-Myb,* but HepG2 cells do not, therefore *c-Myb* transcription factor silencing was performed only in MCF7 cells. In the presence of *c-Myb*, fulvestrant up-regulated the luciferase activity of *UGT1A4* promoter expressing the common alleles 1.5-fold more than the promoter with the variant alleles (p < 0.01, Figure 5). When *c-Myb* was silenced, no fulvestrant-induced up-regulation in luciferase activity was detected (Figure 5). *c-Myb* appears to affect fulvestrant-induced *UGT1A4* promoter activity in the promoter with common alleles, but variation in the *UGT1A4* promoter inhibits activity and the effect of *c-Myb* binding.

### Induction of multidrug resistance-associated protein by fulvestrant

MRPs have been characterized as apical glucuronide export pumps. (Cui et al. 1999;Munzel et al. 1999). In order to investigate regulation by fulvestrant, *MRP* expression levels were analyzed in MCF7 and HepG2 cell lines. Cells were treated with 10nM fulvestrant, and *MRP1, MRP2* and *MRP3* expression levels were measured at various time points. Gene expression was up-regulated for *MRP1, MRP2* and *MRP3,* and *expression* peaked at 48 hours in both cell lines. Thus, *MRP*s and *UGT1A4* were coordinately induced by fulvestrant. *UGT1A4* mRNA levels correlate with *MRP1* (r = 0.972; p < 0.05), *MRP*2 (r = 0.906; p<0.01) and *MRP*3 (r = 0.983; p < 0.05) expression levels in MCF7 cells. *UGT1A4* mRNA levels correlate with *MRP*2 (r = 0.948; p < 0.05) and *MRP*3 (r = 0.967; p < 0.05) expression in HepG2 cells (*MRP1*is not expressed in HepG2; Table 1).

## Discussion

Fulvestrant is effective in treating tamoxifen resistant ER-positive metastatic breast cancer tumors (Robertson et al. 2001). Recently, clinical trials investigating the utility of adding fulvestrant to other therapeutics have not been shown to affect cytochrome P450-mediated metabolism of either of the applied drugs (Robertson et al. 2004;Hiscox et al. 2009). Effects of co-administration on phase II metabolism and drug transporter genes, however, have not been explored. Metabolic biotransformation of endogenous and exogenous compounds renders lipophilic molecules more soluble in water, allowing their excretion to bile, urine or feces. Altered rates of metabolism can influence the systemic availability and residence time of xenobiotics, and hence affect xenobiotic toxicity or therapeutic effect. It is well established that certain xenobiotics induce the expression of specific Phase I and II metabolizing enzymes (Catania et al. 2004;Bock et al. 2000;Cummings et al. 2002). Better understanding of simultaneous regulation of metabolism and disposition may help to prevent these undesirable effects. In this study, the effects of fulvestrant treatment on the phase II enzyme UGT1A4 and the *MRP* family of drug transporters in ER + MCF7 and HepG2 cells were examined.

The high sensitivity of MCF7 and HepG2 cells towards fulvestrant (Woode et al. 2012;Osborne et al. 2004;Wakeling and Bowler 1987;Hu et al. 1993;Catania et al. 2004) limits time in *in vitro* experiments at higher doses. In order to perform experiments at the highest effective dose for extended times, cells were pre-treated with 1nM fulvestrant for 30 days. This allowed for the experimental conditions used in this paper. Concentration and time experiments showed that treatment with 10 nM fulvestrant for 48 hours significantly increased *UGT1A4* expression in both cell lines.

Up regulation of *UGT1A4* mRNA correlates with UGT1A4 protein expression, demonstrating that UGT1A4 protein expression is regulated at least partially by transcription. In previous studies, human liver microsomes with higher expression of UGT1A4 exhibited increased glucuronidation of anastrozole (Edavana et al. 2013). To determine if fulvestrant-induced increases of UGT1A4 expression had a similar effect in MCF7 and HepG2 cell lines, anastrozole glucuronidation assays were performed after fulvestrant treatment to simulate co-treatment. UGT1A4 expression correlated with anastrozole glucuronidation, indicating that up regulation of UGT1A4 by fulvestrant has an effect on co administration of drugs that are substrates of UGT1A4.

The increase in UGT1A4 upon fulvestrant treatment is thought to be mediated through the ERα pathway. When *ERα* was silenced in MCF7 and HepG2 cell lines, fulvestrant treatment no longer up regulated *UGT1A4* expression, confirming that the ERα pathway has a role in *UGT1A4* up regulation. There is no correlation between *UGT1A4* expression and activity with ERα protein levels; however, there is correlation with ERα mRNA, indicating that there are other mechanisms or pathways co-regulating *UGT1A4* expression upon fulvestrant treatment.

*UGT1A4* promoter SNPs may also play a role in fulvestrant-induced UGT1A4 up regulation. Glucuronidation activities in different human tissues have been shown to exhibit a high degree of variation (Edavana et al. 2013;Shipkova et al. 2001;Strassburg et al. 2000). One explanation is the presence of SNPs within the coding regions of *UGT1A4* genes that may lead to quantitative or qualitative alterations of specific catalytic activities (Ehmer et al. 2004;Edavana et al. 2013). Previous studies have revealed 3 SNPs located upstream of the ATG codon at -163, -217, and -219 (Edavana et al. 2013;Saeki et al. 2005;Erichsen et al. 2008;Benoit-Biancamano et al. 2009). In this study, reporter gene experiments show that *UGT1A4* transcription after fulvestrant treatment is significantly reduced with either the -163A or the -219T variant genotype compared to those with the common -163G or -219C genotype in MCF7 and HepG2 cell lines. In HepG2 cells, there was also a statistically significant increase in *UGT1A4* expression with the -217G variant genotype. The genotype with all common alleles (-163G, -217 T and -219C) appears to sustain full activity of the *UGT1A4* gene promoter fragment, but the genotype with variant alleles reduced the activity by 40-60% in both cells lines. Combined, these data demonstrate that small variations in the *UGT1A4* gene promoter region alter constitutive expression of *UGT1A4* upon fulvestrant treatment. These findings may be relevant for co-administration of drugs that areUGT1A4 substrates such as tamoxifen, anastrozole, clozapine and lamotrigine. Genetic variants leading to constitutive expression and that alter the ability to respond to physiological inducers contribute to inter-individual variability in glucuronidation capacity. Therefore, pharmacogenetic risk associated with these variants should be considered in clinical studies.

Another factor related to the up regulation of *UGT1A4* by fulvestrant treatment could be through changes in the binding capacity of transcription factors due to the presence of promoter SNPs in *UGT1A4* (Quintana et al. 2011). *In silico* analysis with the is-rSNP program revealed that the -163A variant is associated with changes in the *c-Myb* transcription factor binding site (Quintana et al. 2011). c-Myb is an oncogene that is up regulated in breast cancer cells, and has been associated with estrogen response in breast cancer. Silencing *c-Myb* in MCF7 cell lines (*c-Myb* is not expressed in HepG2 cells) reduced fulvestrant induced luciferase activity of the promoter with all common alleles by 1.5 fold. There was no change in fulvestrant induced luciferase expression in the promoter with all variant alleles. This is the first time that c-Myb has been reported to have a regulatory effect on the phase II metabolizing gene UGT1A4.

Interplay between transporters and drug-metabolizing enzymes has been postulated to have a major role in determining a drug’s absorption and disposition (Wacher et al. 1995;Custodio et al. 2008;Pang et al. 2009). Phase II enzymes are localized with their transport systems, and both are induced by the same compounds, suggesting a correlated action (Bock et al. 2000). The transporter genes *MRP1*, *MRP2*, and *MRP3* are expressed in MCF7 cells, but only *MRP2* and *MRP3* are expressed in HepG2 cells (Ros et al. 2003). This study demonstrated that fulvestrant-induced *UGT1A4* expression correlated with increased expression of *MRP*1 in MCF7 cells, and of *MRP*2 and *MRP*3 in MCF7 and HepG2 cells. The present study is the first report of *UGT1A4* and *MRP1*, *MRP*2 and *MRP*3 being coordinately induced by fulvestrant.

## Conclusion

This data suggest that the *UGT1A4*-inducing activity of fulvestrant is probably mediated by *ERα*, *c-Myb* and promoter SNPs. This data also demonstrates that transporter genes *MRP1, MRP2* and *MRP3* are also induced by fulvestrant, suggesting they may play a role in drug disposition in co-treatments. The clinical application of pharmacogenomics in cancer treatment will therefore require more detailed information concerning the functional effects of genetic variants in drug metabolizing enzymes and drug transporters.

## Abbreviations

ER: Estrogen receptor
MRPs: Multidrug resistance-associated proteins
UGT1A4: UDP glucuronosyltransferase 1A4
GSTs: Glutathione S-Transferases
SULTs: Sulfotransferases
SERMs: Selective estrogen receptor modulators
UDPGA: UDP glucuronic acid
TED: Tris-EDTA-diethyldithiocarbamic
PWM: Position weight matrix.
